# Depressive and Anxiety Symptoms, Defense Mechanisms, and Mentalized Affectivity in Individuals with Myocardial Infarction: An Empirical Investigation

**DOI:** 10.3390/bs15040528

**Published:** 2025-04-14

**Authors:** Gianluca Cruciani, Gabriele Lo Buglio, Annalisa Tanzilli, Marianna Liotti, Matteo Scalzeri, Gaetano Tanzilli, Federica Galli, Vittorio Lingiardi

**Affiliations:** 1 Department of Systems Medicine, University of Rome Tor Vergata, 00133 Rome, Italy; gianluca.cruciani@uniroma2.it; 2 Department of Dynamic and Clinical Psychology, and Health Studies, Faculty of Medicine and Psychology, Sapienza University of Rome, 00185 Rome, Italymatteo.scalzeri@uniroma1.it (M.S.);; 3 Department of Genetics & Molecular Biology, Sapienza University of Rome, 00161 Rome, Italy; 4 Department of Clinical Internal, Anesthesiologic and Cardiovascular Sciences, Sapienza University of Rome, 00185 Rome, Italy; gaetano.tanzilli@uniroma1.it

**Keywords:** myocardial infarction, defense mechanisms, anxiety, depression, mentalized affectivity

## Abstract

Myocardial infarction (MI) is associated with emotional distress, depression, and anxiety. Defense mechanisms and mentalized affectivity play key roles in affective regulation; however, their roles in individuals with MI remain underexplored. This study examined the relationship between depressive and anxiety symptoms, defensive functioning, and mentalized affectivity in MI patients. Sixty-seven patients with MI and 80 healthy controls completed the DSM-5 Self-Rated Level 2 Cross-Cutting Symptom Measures—Depression and Anxiety—Adult, the Defense Mechanisms Rating Scales-Self-Report-30, and the Brief-Mentalized Affectivity Scale. Multivariate analyses were used to compare the MI and healthy groups, while correlations and mediation models were used to evaluate associations between variables within the MI group. Compared to controls, patients with MI exhibited more severe depressive and anxiety symptoms, maladaptive defenses, and lower levels of mentalized affectivity. Within the MI group, the severity of depressive and anxiety symptoms was associated with immature defensive functioning and poorer abilities to regulate affects. Notably, the relationship between worse defensive functioning and severe depressive and anxiety symptoms was mediated by the capacity to process emotions. The mediating role of this specific dimension of mentalized affectivity emphasizes the potential of affect regulation as a target for tailored psychosocial interventions aimed at improving clinical outcomes.

## 1. Introduction

With an estimated 17.9 million deaths per year, cardiovascular diseases (CVDs)—in particular acute coronary syndromes (ACSs)—represent the leading cause of mortality worldwide (World Health Organization, 2023) and significantly contribute to determining severe kinds of disability. ACSs are a group of clinical conditions sharing a pathophysiological basis characterized by atherosclerotic plaque rupture or erosion, accompanied by varying degrees of thrombosis and, in some cases, distal embolization, causing myocardial underperfusion (Hamm et al., 2011).

Myocardial infarction (MI) is one of the most serious CVDs and is associated with high mortality rates globally (Moraes-Silva et al., 2017). According to the fourth universal definition of MI (Thygesen et al., 2019), this pathological condition is a specific form of myocardial injury. Diagnosis requires evidence of a rise and/or fall in cardiac troponin values, with at least one value exceeding the 99th percentile of the upper reference limit, accompanied by at least one of the following clinical features: symptoms of myocardial ischemia, new ischemic electrocardiographic changes, pathological Q waves, imaging evidence of myocardial damage or motion abnormalities, or angiographic evidence of a coronary thrombus. MI can be categorized based on electrocardiogram changes into ST-segment elevation myocardial infarction (STEMI) and non-ST-segment elevation myocardial infarction (NSTEMI), which also corresponds to the extent of blood supply reduction (Bhatt et al., 2022; Collet et al., 2021; Ibanez et al., 2018). A large-scale meta-analysis reports that the prevalence of MI is 9.5% in individuals more than 60 years of age and 3.8% in those less than 60 years of age (Salari et al., 2023).

Notably, individuals with MI frequently suffer from mental health problems, such as depression and anxiety, which are highly prevalent in this population and may impact short- and long-term outcomes. Clinical depression affects more than one-fourth of individuals with MI (Feng et al., 2019), with research linking post-infarction depression to an increased risk of new cardiovascular events and all-cause mortality (Meijer et al., 2013). Similarly, moderate-to-severe anxiety symptoms are present in over one-third of individuals with MI (Lian et al., 2022), with evidence highlighting the associations between anxiety and poor clinical outcomes, including short-term complications, adverse long-term prognosis, major cardiac events, and mortality (Wen et al., 2021).

Despite the use of heterogeneous tools, the prevalence rates of depression and anxiety in individuals with MI are more than 6 and 8 times higher than those observed in the general population (World Health Organization, 2022). The critical role of depressive and anxiety symptoms in individuals with MI supports the need for research on the psychological mechanisms underlying these mental health problems, which may inform the development of tailored psychosocial interventions and improve clinical outcomes. This research focus aligns with a recent scientific statement from the American Heart Association (Levine et al., 2021), which recognizes the close relationship between psychological and cardiovascular health and highlights the importance of research in promoting psychological health.

Several studies have pointed out a strong relationship between depression and anxiety and defense mechanisms in different (clinical and non-clinical) populations (e.g., DeFife & Hilsenroth, 2005; Di Giuseppe et al., 2024; Fiorentino et al., 2024; Høglend & Perry, 1998; Olson et al., 2009). From this perspective, depression and anxiety in patients with MI could be conceptualized as the product of ineffective strategies to manage and cope with negative feelings, which in turn may lead to new cardiac episodes and a heightened risk of rehospitalization. In this vein, defense mechanisms (or, simply, “defenses”) should be mentioned as fundamental psychological capacities essential for dealing with stressful life situations, including MI. Defenses are defined as primarily automatic psychological processes that protect individuals from painful emotions, internal conflicts, and challenging events (American Psychiatric Association [APA], 2000; Di Giuseppe & Perry, 2021). Within the hierarchical model operationalized by Perry through the Defense Mechanisms Rating Scale (DMRS; Perry, 1990), they are conceptualized along a continuum from maturity to immaturity. Mature defenses, such as self-assertion and sublimation, are linked to adaptive responses, high awareness, and resilience, whereas immature defenses, such as acting out and projective identification, are associated with maladaptive responses, low awareness, and psychological distress (Di Giuseppe et al., 2024; Prout et al., 2022; for a full description of defensive categories and defense levels, see Table S1 in the Supplementary Materials). Notably, preliminary research on individuals with MI identified a positive correlation between immature defenses and depressive symptoms and negative associations between mature defenses and both depressive and anxiety symptoms (Korkmaz et al., 2022); however, the complex interplay between defenses and mental health symptoms in this population has never been explored.

Notably, research has indicated that MI profoundly impacts the lives of those affected, often triggering significant emotional distress (Liljeroos et al., 2023; Svedlund & Danielson, 2004). Emotion regulation difficulties have long been associated with higher depressive and anxiety symptoms in several populations (Kraiss et al., 2020; Schäfer et al., 2017), including patients with CVDs (Chauvet-Gelinier & Bonin, 2017; Cheng et al., 2022; Cruciani et al., 2023; Javadi et al., 2017; Lazzeroni et al., 2022). Particularly, it was stressed that the experience of a cardiac episode may lead individuals to escape their negative feelings of depression and anxiety, which in turn may determine long-term health problems due to persistent dysfunctional emotional regulation, especially through avoidance strategies (Compare et al., 2014; Wierenga et al., 2022). Within this framework, mentalized affectivity (Jurist, 2018) may represent a key psychological capacity relevant to emotion regulation. It has primarily been studied in psychological research but has been overlooked in MI studies. This dimension refers to the ability to identify (i.e., recognize and reflect on emotions and their influences), process (i.e., modulate and differentiate complex emotions), and express (i.e., outwardly or inwardly) emotions through the lens of autobiographical memory (Cruciani et al., 2024; Greenberg et al., 2017; Liotti et al., 2021). Mentalized affectivity connects emotional recognition, expression, and regulation, while emphasizing critical aspects of psychological well-being, including empathy, curiosity, cognitive flexibility, and self-awareness. It promotes the development of adaptive self-representations by integrating emotional regulation with the re-evaluation of emotions in the context of past and present experiences (Jurist, 2018). It also shows a strong association with defense functioning (e.g., Tanzilli et al., 2021). Despite the critical role played by defense mechanisms and mentalized affectivity in affective regulation processes, to date, no study has investigated the relationship between depression, anxiety, and these psychological variables in MI patients.

In light of these promises, the present study had the following objectives:

(1) to evaluate the differences between patients with MI and healthy controls regarding depressive and anxiety symptomatology, defensive functioning, and mentalized affectivity. In line with previous studies (Lian et al., 2022; Mensour et al., 2022), it was hypothesized that higher levels of depressive and anxiety symptoms would characterize the MI patient group. Additionally, although specific literature on this topic is rather scarce, patients with MI would display less adaptive defensive functioning and lower mentalized affectivity levels than healthy controls, as shown by previous research on coping and emotion regulation strategies (Colovic et al., 2016; Fiorentino et al., 2024; Liljeroos et al., 2023);
(2) to investigate the associations between depressive and anxiety symptoms, defensive functioning, and mentalized affectivity within a sample of MI patients. More specifically, according to some empirical contributions in the field (e.g., Fiorini Bincoletto et al., 2025; Di Giuseppe et al., 2024; Tanzilli et al., 2021), it was hypothesized that depressive and anxiety symptoms would be associated with worse global defensive functioning and lower abilities in the realms of mentalized affectivity.
(3) to verify in an exploratory analysis whether the associations between defensive functioning and both depressive and anxiety symptoms could be mediated by mentalized affectivity abilities in MI patients. Consistent with the aforementioned literature, it was hypothesized that impaired mentalized affectivity would partially mediate the association between poor overall defensive functioning and severe levels of depression and anxiety symptom patterns.

## 2. Materials and Methods

### 2.1. Participants

The clinical group comprised 67 patients (16 females, mean age = 61.60 ± 9.77 years) who were admitted for an MI episode to the Department of Clinical Internal, Anesthesiologic, and Cardiovascular Sciences of Sapienza University of Rome between November 2022 and August 2024. Patients were included after a diagnosis of acute MI was made following the guidelines of the European Society of Cardiology for managing acute myocardial infarction in patients presenting with STEMI and NSTEMI (Collet et al., 2021; Ibanez et al., 2018). The exclusion criteria for this group were: age below 18 years; not fluent in Italian; having active cancer, liver cirrhosis, or chronic infectious or autoimmune disease; and being incapable of understanding the study’s instructions.

Eighty healthy participants matched for gender and age (25 females, mean age = 59.00 ± 8.46 years; gender distribution comparison: χ^2^ = 0.985; *p* = 0.321; mean age comparison: t(145) = 1.728; *p* = 0.086) were recruited to compose the control group. Control group recruitment followed a non-random sampling from the general population based on advertisements shared on social media by the research group and word of mouth. Exclusion criteria for the control group were: previous or current CVDs; being active smokers; having a Body Mass Index (BMI) ≥ 30; age below 18 years; being not fluent in Italian; having active cancer, liver cirrhosis, chronic infectious or autoimmune disease; being incapable of understanding the study’s instructions.

### 2.2. Procedure

An online survey using SurveyMonkey was developed and employed to collect data for the present study. The survey included sociodemographic characteristics, clinical information, and four self-reported questionnaires for the evaluation of the MI sample’s depressive and anxiety symptoms, defensive functioning, and mentalized affectivity. Completing the survey required about 40 min. All data collected were anonymized using alphanumeric codes to prevent participant identification. For what specifically concerned the MI group, the survey was administered between 3 and 5 days after hospitalization due to the MI event, before they were discharged.

Participation in the study was voluntary, and all participants were free to withdraw from the study at any time without any consequences. All participants provided written informed consent before participating in the study. The current study conforms to the World Medical Association Declaration of Helsinki of 1975, as revised in 2008, and received approval from the ethics committee of the Department of Dynamic and Clinical Psychology and Health Studies, Sapienza University of Rome (Protocol Number 0000148/2022).

### 2.3. Measures

*Sociodemographic and clinical information:* Sociodemographic (i.e., gender, age, marital status, and education) and clinical information (i.e., BMI, smoking habits, alcohol consumption, previous heart diseases, dyslipidemia, insulinemia, pulmonary edema, transient ischemic attacks, renal failure, diabetes, chronic obstructive pulmonary disease, sleep apnea, and neoplasms) were collected using an ad hoc questionnaire.

*Depressive and anxiety symptoms:* Depressive and anxiety symptomatology were assessed using the DSM-5 Self-Rated Level 2 Cross-Cutting Symptom Measures—Depression and Anxiety—Adult (American Psychiatric Association, 2013). Both the depression (DSM-5-D) and anxiety (DSM-5-A) scales evaluate how often the person has been affected by symptoms over the previous 7 days on a 5-point Likert scale from 0 (“never”) to 4 (“always”), across 8 and 7 items, respectively. In the present study, the total raw score of each scale was converted to standardized T-scores, which were used as overall indices of depressive and anxiety symptomatology. The DSM-5-D and DSM-5-A scales showed excellent levels of reliability (Streiner, 2003), with Cronbach’s alpha values of 0.93 and 0.92, respectively.

*Defensive functioning:* Participants’ defensive functioning was assessed using the Defense Mechanisms Rating Scales-Self-Report-30 (DMRS-SR-30) (Di Giuseppe et al., 2020), a self-report questionnaire derived from the observer-rated Defense Mechanisms Rating Scales Q-sort version (Di Giuseppe et al., 2014). The questionnaire comprises 30 items on a Likert scale ranging from 0 (“not at all”) to 4 (“very often/much”), which describe the hierarchy of defense mechanisms developed by Perry (1990). The defense hierarchy ranges from the most to the least adaptive: (a) High Adaptive (i.e., affiliation, altruism, anticipation, humor, self-assertion, self-observation, sublimation, and suppression), (b) Obsessional (i.e., undoing, intellectualization, and isolation of affects), (c) Neurotic (i.e., repression, dissociation, reaction formation, and displacement), (d) Minor image-distorting (i.e., idealization of self and others’ images, devaluation of self and others’ images, and omnipotence), (e) Disavowal (i.e., denial, rationalization, projection, and autistic fantasy), (f) Major Image-Distorting (i.e., splitting of self and others’ images, and projective identification), and (g) Action defenses (i.e., acting out, passive aggression, and help-rejecting complaining). The DMRS-SR-30 provides a score for each of the seven levels of the hierarchy of defense mechanisms, as well as a total score regarding overall defensive functioning (ODF). The scale showed good reliability (Streiner, 2003), with Cronbach’s alpha values ranging from 0.67 to 0.80.

*Mentalized affectivity.* Mentalized affectivity was evaluated using the Brief-Mentalized Affectivity Scale (B-MAS; Greenberg et al., 2021; Liotti et al., 2021). The B-MAS is a self-reported questionnaire assessing a form of affect regulation that entails revaluing, not just modulating, affective experience (Jurist, 2018) through 12 items on a Likert scale ranging from 1 (“strongly disagree”) to 7 (“strongly agree”). Three fundamental components of mentalized affectivity are as follows: “Identifying emotions”, which pertains to the ability to label one’s emotions as well as comprehend their deep meaning; “processing emotions”, which implies the ability to modulate the intensity of affects, working through and managing them within their social-affective context; and “expressing emotions”, which concerns the capacity to communicate one’s own feelings to others effectively. These three scales of the B-MAS showed good reliability (Streiner, 2003), obtaining the following Cronbach’s alpha values: identifying emotions, α = 0.67; processing emotions, α = 74; and expressing emotions, α = 0.70.

### 2.4. Statistical Analyses

All analyses were performed using JAMOVI version 2.4.11 and the jAMM statistical package (Gallucci, 2021). Preliminary group differences in sociodemographic and clinical information were first run to provide a general sample description. Three multivariate analyses of variance (MANOVAs) were performed to evaluate group differences between MI patients and healthy controls in terms of depressive and anxiety symptoms, defensive functioning, and mentalized affectivity. Specifically, the DSM-5-D and DSM-5-A scores were used as dependent variables in the first MANOVA. The seven DMRS-SR-30 defense level scores (i.e., High Adaptive, Obsessional, Neurotic, Minor Image-Distorting, Disavowal, Major Image-Distorting, and Action) were used as the dependent variables in the second MANOVA. Additionally, a univariate ANOVA was performed considering the DMRS-SR-30 overall defensive functioning (ODF) as the dependent variable to distinguish the overall defensive functioning between the clinical and healthy groups. Finally, the B-MAS scales for identifying, processing, and expressing emotions were included as dependent variables in the final MANOVA.

To explore possible associations between depressive and anxiety symptoms, defensive functioning, and mentalized affectivity in the MI group, bivariate correlations (Pearson’s *r*, 2-tailed) were calculated between the DSM-5-D and DSM-5-A scores, the DMRS-SR-30 defense level scores and ODF, and the B-MAS scales.

To verify the potential mediating role of mentalized affectivity (assessed using the B-MAS) in the association between defensive functioning (i.e., ODF evaluated using the DMRS-SR-30) and psychopathological symptoms (assessed using the DSM-5-D and the DSM-5-A) among MI patients, two sets of General Linear Model (GLM) mediation models were performed. In both sets, ODF was the predictor variable and B-MAS was the mediator variable. In the first set, depression levels measured by the DSM-5-D were the criterion variable, whereas in the second set, anxiety symptoms measured by the DSM-5-A were the criterion variable. GLM mediation models were implemented according to the criteria proposed by Baron and Kenny (1986).

## 3. Results

Table S2 in the Supplementary Materials displays the descriptives and comparisons between patients with MI and healthy controls on sociodemographic and clinical variables.

Concerning group differences in depressive and anxiety symptomatology, the MANOVA with Group (MI patients vs. healthy controls) as the independent variable and DSM-5-D and DSM-5-A scores as the dependent variables showed significant results (Wilks’s λ = 0.805; *F*(2, 144) = 17.406; *p* < 0.001; ηp^2^ = 0.195). In detail, patients with MI were characterized by significantly higher levels of depression, *F*(1, 146) = 24.447; *p* < 0.001; ηp^2^ = 0.144, and anxiety symptoms, *F*(1, 146) = 34.495; *p* < 0.001; ηp^2^ = 0.192, than controls.

Regarding defensive functioning, the MANOVA with Group as the independent variable and the seven DMRS-SR-30 defense levels (i.e., High Adaptive, Obsessional, Neurotic, Minor Image-Distorting, Disavowal, Major Image-Distorting, and Action) scores as dependent variables showed significant findings, Wilks’s λ = 0.857; *F*(7, 139) = 3.309; *p* = 0.003; ηp^2^ = 0.143. Particularly, the MI patient group was characterized by lower High Adaptive defense level scores, *F*(1, 146) = 10.928; *p* = 0.001; ηp^2^ = 0.070, and higher Neurotic level, *F*(1, 146) = 16.046; *p* < 0.001; ηp^2^ = 0.100, and Disavowal level scores, *F*(1, 146) = 10.787; *p* = 0.001; ηp^2^ = 0.069, than healthy controls. Additionally, ANOVA with Group (MI patients vs. healthy controls) as the independent variable and the DMRS-SR-30 ODF as the dependent variable showed lower scores for the MI group, *F*(1, 146) = 9.422, *p* = 0.003; ηp^2^ = 0.061.

Finally, regarding mentalized affectivity, the MANOVA with Group as the independent variable and B-MAS scales (i.e., identifying, processing, and expressing emotions) as the dependent variables yielded significant results, Wilks’s λ = 0.784; *F*(3, 143) = 13.157; *p* < 0.001; ηp^2^ = 0.216. Specifically, MI patients displayed lower scores in all three dimensions of mentalized affectivity: identifying emotions, *F*(1, 146) = 30.169; *p* < 0.001; ηp^2^ = 0.172; processing emotions, *F*(1, 146) = 7.024; *p* = 0.009; ηp^2^ = 0.046; and expressing emotions, *F*(1, 146) = 17.527; *p* < 0.001; ηp^2^ = 0.108) than healthy controls. All MANOVA findings (including descriptive statistics) are presented in Table 1.

Table 2 reports the full matrix of correlations between depressive and anxiety symptoms, defensive functioning (taking into account all seven levels of the DMRS-SR-30 and ODF), and mentalized affectivity (including all the scales) among MI participants. Notably, DSM-5-D showed stronger and significantly positive correlations with DMRS-SR-30 Neurotic, Disavowal, and Major Image-Distorting defense levels, whereas it was significantly negatively related to the High Adaptive defense level and ODF. Similarly, DSM-5-A scores were strongly and significantly positively associated with the DMRS-SR-30 Obsessional, Neurotic, and Action defense levels and negatively related to Highly Adaptive defenses and ODF. It is important to highlight that the DSM-5-D and DSM-5-A were significantly correlated with the B-MAS processing emotion scale.

Following Baron and Kenny’s recommendations (Baron & Kenny, 1986), all variables included in the mediation models must be significantly associated with each other. Thus, two GLM mediation models were performed to assess the potential mediating role of the processing emotion scale (assessed using the B-MAS) in the association between ODF (assessed using the DMRS-SR-30) and psychopathological symptoms (assessed using the DSM-5-D and DSM-5-A) in patients with MI.

**Figure 1:**
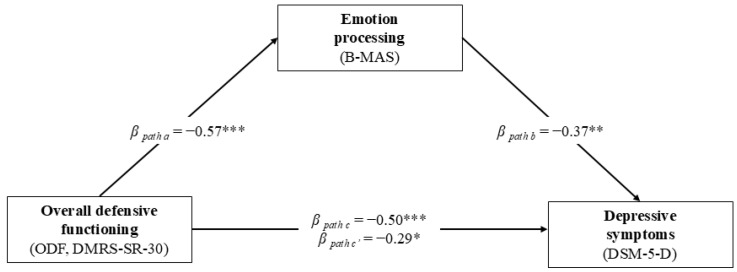
Graphical representation of the GLM mediation model assessing the direct and indirect effects of overall defensive functioning (assessed using the DMRS-SR-30) on depressive symptoms (assessed using the DSM-5-D) through emotion processing (assessed using the B-MAS) in patients with MI (N = 67). * *p* ≤ 0.05. ** *p* ≤ 0.01. *** *p* ≤ 0.001.

In the first model depicted in Figure 1, the ODF was the predictor variable, the B-MAS processing scale was the mediator variable, and the DSM-5-D was the criterion variable. The results are presented in Table 3. Overall, the GLM mediation model showed that ODF had a significant indirect effect on depressive symptoms through the pathway of the B-MAS processing scale (*β* = −0.208; *z* = −2.663; *p* = 0.008), accounting for about 42% of the total variance.

**Figure 2:**
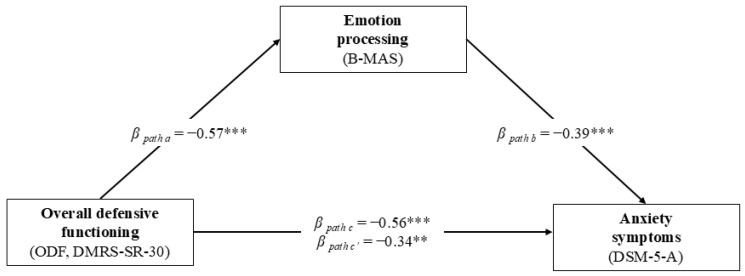
Graphical representation of the GLM mediation model assessing the direct and indirect effects of overall defensive functioning (assessed with the DMRS-SR-30) on anxiety symptoms (assessed with the DSM–5–A) through emotion processing (assessed with the B-MAS) in patients with MI (N = 67). ** *p* ≤ 0.01. *** *p* ≤ 0.001.

Similarly, in the second mediation model shown in Figure 2, the ODF was the predictor variable, the B-MAS processing scale was the mediator variable, and the DSM-5-A was the criterion variable. As reported in Table 4, the GLM mediation model showed that ODF had a significant indirect effect on anxiety symptoms through the pathway of the B-MAS processing scale (*β* = −0.224; *z* = −2.959; *p* = 0.003), accounting for about 40% of the total variance.

## 4. Discussion

The current study investigated the relationship between anxiety and depressive symptomatology, defensive functioning, and mentalized affectivity in the MI patient group, attempting to shed light on the dynamic processes underlying the most prevalent psychopathological patterns in this specific ACS population (e.g., Levine et al., 2021). The results showed that patients with MI presented severe depressive and anxiety symptoms, along with low defensive functioning characterized by the adoption of primarily maladaptive defenses and poor capacities for identifying, processing, and expressing emotions within the mentalized affectivity realms (Jurist, 2018). Overall, this research indicates that patients with MI seem to suffer from clinically relevant levels of depression and anxiety, which could contribute to an increased risk of adverse cardiovascular events and mortality (Carney et al., 2003). Moreover, these psychopathological patterns were strongly related to immature defensive functioning and lower abilities to regulate one’s own affect.

The first objective was to compare patients with MI and healthy controls with respect to levels of depression and anxiety, defensive functioning, and the quality of mentalized affectivity. Consistent with the hypotheses and empirical literature in the field (e.g., Carney et al., 2003; Thombs et al., 2008), depressive and anxiety symptoms were highly prevalent in the MI group. Over the years, research on mind-heart interaction has provided strong evidence that mental health is substantially related to cardiac dysfunction, highlighting significant clinical implications. Anxiety and depression, as well as the mechanisms through which individuals regulate their emotions and cope with stressful situations, are believed to impact both the development and progression of CVDs and their associated risks (Iso et al., 2002; Rosengren et al., 2004). The pathways underpinning these connections are complex, as they comprise a combination of behavioral and physiological factors. From a behavioral standpoint, depressive and anxiety symptoms may contribute to a lower quality of life in patients with MI (Kang et al., 2017), which is often associated with fatigue, social isolation, lower self-care, and diminished daily activities, and, consequently, to the development or maintenance of CVD risk factors, including medication non-adherence, lack of exercise, and obesity (Pietrzykowski et al., 2020; Wang et al., 2016). Additionally, patients may resort to dysfunctional behavioral patterns to cope with and regulate their negative feelings, such as smoking or alcohol assumption, further contributing to the reoccurrence of CVDs (Rørholm Pedersen et al., 2016). From a physiological perspective, depression is associated with MI risk through a series of pathophysiological mechanisms, including dysfunction in serotoninergic regulation of cardiac parasympathetic activity, gut microbiota imbalance, alteration of the hypothalamus–pituitary–adrenal axis regulation of the sympathetic system, exosome maladjustment, and enhancement of inflammatory processes (Yang et al., 2021). Anxiety is suggested to be related to several pathophysiological processes in patients with MI, including arrhythmic mechanisms (e.g., tachycardia) and alterations in autonomic nervous system balance (e.g., reduced baroreflex cardiac control and reduced heart rate variability) (Roest et al., 2010). Similar pathways appear to be involved in the association between MI and emotion dysregulation and maladaptive coping strategies. For instance, neural functional alterations in brain areas involved in emotion regulation and coping strategies (e.g., amygdala, anterior cingulate cortex, medial prefrontal cortex, and insula) were linked to impairments in heart rate, heart rate variability, cardiac contractility, baroreflex sensitivity, blood pressure, and inflammatory responses via neuroanatomical connections between cortical and limbic regions and peripheral physiology (Kraynak et al., 2018).

Our findings corroborate and expand the evidence on the high prevalence and severity of depressive and anxiety symptoms in individuals with MI, further highlighting the need for interventions for such common mental health issues. The psychopathological patterns in this population can be considered modifiable risk factors for a wide range of poor outcomes, including mortality (Mal et al., 2019). This approach is consistent with a scientific statement from the American Heart Association (Lichtman et al., 2014) that explicitly frames depression as a risk factor for recurrent cardiovascular events in individuals who survive ACS. Similarly, meta-analytic evidence highlights the association between anxiety and the risk of mortality in individuals with MI (Wen et al., 2021). Notably, an increasing body of evidence emphasizes the importance of integrating subjective patient-reported outcomes, such as depressive and anxiety symptoms assessed using self-report tools, with objective data from biomedical assessments (Norekvål et al., 2010). Utilizing both approaches can provide a more comprehensive clinical understanding and aid in identifying individuals at the highest risk of adverse outcomes (Krumholz et al., 2005).

In line with the second aim, the study showed that individuals with MI who suffered from severe depressive and anxiety symptoms exhibited less adaptive overall defensive functioning and meaningful impairments in mentalized affectivity. These results suggest increased difficulties in coping with and managing painful, stressful, or emotionally charged events in this population (Svedlund & Danielson, 2004; Thompson et al., 2002). Potentially, challenging circumstances may activate intense emotional reactions in patients with MI, which are complex to recognize, regulate, and express in an adaptive way, leading them to use non-mature defensive strategies, especially those that suppress awareness of unacceptable thoughts, ideas, or motives (i.e., disavowal defenses) (Di Giuseppe & Perry, 2021; Liotti et al., 2021). Consistent with evidence from prognostic research (Chiavarino et al., 2012; Denollet & Brutsaert, 2001; Roest et al., 2010; Van Beek et al., 2016), our findings support the need to assess mental health symptoms and their subjective experiences, as well as strategies for recognizing emotions and dealing with difficult life situations. This valuable approach, based on assessing and monitoring specific psychological dimensions, can guide the development of tailored psychosocial interventions aimed at enhancing individual well-being and reducing the risk of physical health complications in both the short and long term.

Consistent with research conducted on individuals with mental health problems (Colovic et al., 2016; Di Giuseppe et al., 2024; Fiorentino et al., 2024), MI patients with high anxiety and depressive symptoms were prone to resorting less to adaptive defenses, showing a wider use of immature defense mechanisms (especially obsessional, neurotic, disavowal, and major image-distorting levels). This result indicates that individuals with MI may tend to cope with challenging events and feelings, keeping greater distance from emotions, blocking full awareness of ideas, refusing to acknowledge aspects of internal experience or external reality, and splitting positive and negative representations of self and others (Di Giuseppe & Perry, 2021; Prout et al., 2022). At the same time, these patients tended to make little use of mature defenses, which are an index of psychological health (Amaslidou et al., 2023; Békés et al., 2024; Di Giuseppe et al., 2018; Hyphantis et al., 2013; Malone et al., 2013). Thus, it is important to promote the development of defensive or coping strategies that can aid in facing high-arousal situations, maintain a heightened awareness of internal experiences, and activate evolutionary resources and resilience (e.g., Babl et al., 2019; da Silva Machado et al., 2023; Di Giuseppe et al., 2024; Di Giuseppe & Conversano, 2022; Tanzilli et al., 2022). Moreover, the study’s findings showed that all abilities of identifying, processing, and expressing emotions in MI patients were positively related to mature defenses and overall defensive functioning, underscoring the close relationship between effective emotional management and the use of adaptive strategies in promoting positive expression and satisfying personal motives without distorting reality. From this perspective, mentalized affectivity may help encompass key aspects of psychological well-being, such as curiosity, empathy, and cognitive flexibility (Jurist, 2018). These qualities can enhance adaptive capacities to address difficulties and challenges linked to MI and its associated psychological burdens.

The present study aimed to explore two mediation models in order to deepen the understanding of the complex associations between lower overall defensive functioning, poor mentalized affectivity, and severe depressive and anxiety symptoms. Interestingly, the results highlighted the critical role of processing emotions in mediating the relationship between defenses and common mental health symptom patterns in the MI population. This result suggests that regulating emotions, modulating their intensity, and working through their complexity represent the key mechanism that is able to clarify the impact of the maladaptive capability of MI individuals to respond and protect themselves from stressors and the severity of depressive and anxiety pathology. This result emphasizes the potentially protective value of processing emotions within the mentalized affectivity domain and supports psychosocial interventions that strengthen the capacity to regulate intense emotions that may emerge from MI. Improving this capacity could improve mental health and prevent adverse outcomes. Future studies with a longitudinal design assessing psychological mechanisms, mental health symptoms, and physical health outcomes are warranted to explore the effects of this component of mentalized affectivity over time.

This study has several strengths, including a matched healthy control group, the assessment of subjectively reported mental health symptoms and adaptive psychological capacities to recognize, modulate, and express emotions, and strategies for coping with stressors. However, some limitations should also be acknowledged, such as the sample size, recruitment of the clinical group from a single center in a Western country, absence of assessment of the mental health status of the patients before the occurrence of MI, and cross-sectional design of the study. In addition, although patients were recruited immediately after the acute MI event and before hospital discharge, the risk of prevalence-incidence bias could not be excluded. In light of these considerations, future research is warranted to address these issues. First, research that includes data from a larger sample size recruited from different centers would help extend the generalizability of the results of the current study. Moreover, cohort studies are important to minimize potential prevalence-incidence bias. Finally, longitudinal studies are required to draw causal relationships among the variables of interest and analyze how individuals’ mental health status might change before and after AMI, further clarifying the possible role of psychological dimensions in the development of acute cardiac events. In the same vein, future research should consider a range of additional social determinants and mental health correlates playing a key role in individuals with MI, including traumatic events in the past year, loneliness, social isolation, pessimism, and work-related stress (Levine et al., 2021; Lo Buglio et al., 2025), as well as biological and genetic predisposing factors.

## 5. Conclusions

The present study revealed a complex interplay between depressive and anxiety symptoms, defensive functioning, and mentalized affectivity in individuals with MI. Research has highlighted the critical role of psychological factors in the management of challenges faced by patients with MI, which contribute to heightened psychological distress and exacerbate adverse cardiac outcomes. In this vein, the present study showed that, after an acute MI event, patients were characterized by more enhanced depressive and anxiety symptoms; this was paired with higher maladaptive defenses and lower levels of mentalized affectivity in the MI group compared with healthy participants. Additionally, the severity of depressive and anxiety symptoms within the MI group was associated with immature defensive functioning and poorer abilities to regulate emotions. Particularly, the present study further analyzed the possible interplay between depressive and anxiety symptoms with defensive functioning and mentalized affectivity, by showing that patients’ ability to process emotions mediated the association between their defensive functioning and psychopathological symptomatology. Notably, the crucial role of mentalized affectivity, in particular the capacity to process emotions, in the relationship between defensive functioning and mental health symptoms underscores the importance of targeting emotion regulation to promote effective mental health intervention protocols. Enhancing the capacity to modulate and work through emotions in patients with MI seems to be essential to avoid potentially more severe psychological and physical outcomes. From a clinical standpoint, this empirical investigation suggests the need to integrate evidence-based psychosocial interventions into cardiac care to strengthen emotional regulation capacities and promote adaptive defense mechanisms. Approaches such as psychodynamic therapy, mindfulness-based interventions, and emotion-focused therapy may be particularly beneficial in fostering emotional awareness, improving coping strategies, and reducing reliance on maladaptive defenses. By addressing and incorporating these psychological dimensions, it is possible to implement personalized interventions that can not only alleviate depressive and anxiety symptoms but also enhance resilience, improve adherence to medical treatment, and potentially reduce the risk of recurrent cardiac events. Future research should examine the longitudinal benefits of such integrated care models, exploring how psychological improvements may “translate” into better cardiac health and quality of life in this vulnerable population.

## Figures and Tables

**Table 1:** Differences between the MI patient group and healthy controls in depressive and anxiety symptoms (assessed using the DSM-5-D and DSM-5-A, respectively), defensive functioning (assessed using the DMRS-SR-30), and mentalized affectivity (assessed using the B-MAS) (N = 147).

	MI Patients (N = 67) Mean ± SD	Healthy Controls (N = 80) Mean ± SD	*F*	*p*	ηp^2^
DSM-5 Self-Rated Level 2 Cross-Cutting Symptom Measures					
DSM-5-D	54.54 ± 8.96	47.23 ± 8.91	24.447	<0.001	0.144
DSM-5-A	57.30 ± 10.49	47.98 ± 8.75	34.495	<0.001	0.192
DMRS-SR-30					
High Adaptive	30.27 ± 11.70	35.34 ± 6.55	10.928	0.001	0.070
Obsessional	10.11 ± 2.97	9.87 ± 2.11	0.346	0.557	0.002
Neurotic	15.56 ± 4.15	13.34 ± 2.45	16.046	<0.001	0.100
Minor Image-Distorting	10.10 ± 2.95	9.97 ± 2.33	0.094	0.760	0.001
Disavowal	14.22 ± 3.15	12.66 ± 2.61	10.787	0.001	0.069
Major Image-Distorting	9.64 ± 2.73	9.35 ± 2.05	0.541	0.463	0.004
Action	10.09 ± 2.83	9.47 ± 2.05	2.398	0.124	0.016
ODF	4.63 ± 0.39	4.79 ± 0.26	9.422	0.003	0.061
B-MAS					
Identifying	3.64 ± 0.97	4.61 ± 1.13	30.169	<0.001	0.172
Processing	3.91 ± 1.41	4.44 ± 1.04	7.024	0.009	0.046
Expressing	3.25 ± 1.24	4.08 ± 1.15	17.527	<0.001	0.108

Note. DMRS-SR-30 = Defense Mechanisms Rating Scales-Self-Report-30; ODF = Overall defensive functioning; B-MAS = Brief-Mentalized Affectivity Scale; DSM-5-D = DSM-5 Self-Rated Level 2 Cross-Cutting Symptom Measures—Depression—Adult; DSM-5-A = DSM-5 Self-Rated Level 2 Cross-Cutting Symptom Measures—Anxiety—Adult.

**Table 2:** Bivariate correlations between depressive and anxiety symptoms (assessed with the DSM-5-D and DSM-5-A, respectively), defensive functioning (assessed with the DMRS-SR-30), and mentalized affectivity (assessed with the B-MAS) among the MI group (N = 67).

	1.	2.	3.	4.	5.	6.	7.	8.	9.	10.	11.	12.	13.
DSM–5 Self-Rated Level 2 Cross-Cutting Symptom Measures													
1. DSM–5–D	1												
2. DSM–5–A	0.74 ***	1											
DMRS-SR-30													
3. ODF	−0.50 ***	−0.56 ***	1										
4. High Adaptive	−0.51 ***	−0.59 ***	0.96 ***	1									
5. Obsessional	0.30 *	0.38 **	−0.60 ***	−0.72 ***	1								
6. Neurotic	0.43 ***	0.52 ***	−0.58 ***	−0.72 ***	0.39 ***	1							
7. Minor Image-Distorting	0.15	0.28 *	−0.44 ***	−0.51 ***	0.36 **	0.22	1						
8. Disavowal	0.43 ***	0.29 *	−0.63 ***	−0.63 ***	0.30 *	0.44 ***	0.05	1					
9. Major Image-Distorting	0.35 **	0.28 *	−0.64 ***	−0.53 ***	0.26 *	0.18	0.05	0.33 **	1				
10. Action	0.21	0.39 ***	−0.71 ***	−0.59 ***	0.40 ***	0.20	0.26 *	0.17	0.26 *	1			
B-MAS													
11. Identifying	−0.16	−0.13	0.36 **	0.37 **	−0.13	−0.25 *	−0.39 ***	−0.32 **	−0.21	−0.09	1		
12. Processing	−0.53 ***	−0.59 ***	0.57 ***	0.61 ***	−0.49 ***	−0.44 ***	−0.26 *	−0.46 ***	−0.25 *	−0.35 **	0.39 ***	0.1	
13. Expressing	−0.18	−0.13	0.29 *	0.30 *	−0.20	−0.16	−0.23	−0.24	−0.13	−0.16	0.23	0.23	1

Note. DMRS-SR-30 = Defense Mechanisms Rating Scales-Self-Report-30; ODF = Overall defensive functioning; B-MAS = Brief-Mentalized Affectivity Scale; DSM-5-D = DSM-5 Self-Rated Level 2 Cross-Cutting Symptom Measures—Depression—Adult; DSM-5-A = DSM-5 Self-Rated Level 2 Cross-Cutting Symptom Measures—Anxiety—Adult. * *p* ≤ 0.05. ** *p* ≤ 0.01. *** *p* ≤ 0.001.

**Table 3:** Indirect and total effects in the GLM mediation model, including the B-MAS processing emotion scale as a mediator in the relationship between the DMRSSR-30 overall defensive functioning and the DSM–5–D depressive symptoms in MI patients (N = 67).

Type	Effect	Estimate	SE	95% CI		*β*	*z*	*p*
				Lower	Upper			
Indirect	DMRS-SR-30—ODF ⇒ B-MAS Processing ⇒ DSM–5–D	−4.761	1.787	−8.381	−1.804	−0.208	−2.663	0.008
Component	DMRS-SR-30—ODF ⇒ B-MAS Processing	2.044	0.360	1.407	2.700	0.570	5.680	<0.001
	B-MAS Processing ⇒ DSM–5–D	−2.329	0.772	−3.781	−0.903	−0.366	−3.015	0.003
Direct	DMRS-SR-30—ODF ⇒ DSM–5–D	−6.557	2.769	−11.528	−1.515	−0.287	−2.368	0.018
Total	DMRS-SR-30—ODF ⇒ DSM–5–D	−11.318	2.443	−15.506	−7.116	−0.495	−4.633	<0.001

Note. DMRS-SR-30 = Defense Mechanisms Rating Scales-Self-Report-30; ODF = Overall defensive functioning; B-MAS = Brief-Mentalized Affectivity Scale; DSM–5–D = DSM–5 Self-Rated Level 2 Cross-Cutting Symptom Measures—Depression—Adult.

**Table 4:** Indirect and total effects in the GLM mediation model, including the B-MAS processing emotion scale as a mediator in the relationship between the DMRSSR-30 overall defensive functioning and the DSM–5–A anxiety symptoms in patients with MI (N = 67).

Type	Effect	Estimate	SE	95% CI		*β*	*z*	*p*
				Lower	Upper			
Indirect	DMRS-SR-30—ODF ⇒ B-MAS Processing ⇒ DSM–5–A	−5.979	2.020	−10.715	−2.019	−0.224	−2.959	0.003
Component	DMRS-SR-30—ODF ⇒ B-MAS Processing	2.044	0.360	1.448	2.753	0.570	5.680	<0.001
	B-MAS Processing ⇒ DSM–5–A	−2.924	0.843	−4.937	−1.100	−0.392	−3.467	<0.001
Direct	DMRS-SR-30—ODF ⇒ DSM–5–A	−9.105	3.025	−15.602	−2.234	−0.340	−3.010	0.003
Total	DMRS-SR-30—ODF ⇒ DSM–5–A	−15.084	2.719	−19.460	−10.517	−0.564	−5.547	<0.001

Note. DMRS-SR-30 = Defense Mechanisms Rating Scales-Self-Report-30; ODF = Overall defensive functioning; B-MAS = Brief-Mentalized Affectivity Scale; DSM–5–A = DSM–5 Self-Rated Level 2 Cross-Cutting Symptom Measures—Anxiety—Adult.

## Data Availability

The data that support the findings of this study are available from the authors upon reasonable request from the corresponding author.

## Supplementary Materials

The following supporting information can be downloaded at: https://www.mdpi.com/article/10.3390/bs15040528/s1, Table S1: Defensive categories and defense levels, adapted from Perry (1990). Table S2: Descriptives and differences between MI patients and healthy controls on sociodemographic and clinical information.

## Abbreviations

The following abbreviations are used in this manuscript:

CVDs	cardiovascular diseases
ACSs	acute coronary syndromes
MI	myocardial infarction
STEMI	ST-segment elevation myocardial infarction
NSTEMI	non-ST-segment elevation myocardial infarction
APA	American Psychiatric Association
DMRS	Defense Mechanisms Rating Scale
BMI	Body Mass Index
DSM-5-D	DSM-5 Self-Rated Level 2 Cross-Cutting Symptom Measures—Depression– Adult
DSM-5-A	DSM-5 Self-Rated Level 2 Cross-Cutting Symptom Measures—Anxiety– Adult
DMRS-SR-30	Defense Mechanisms Rating Scales-Self-Report-30
ODF	overall defensive functioning
B-MAS	Brief-Mentalized Affectivity Scale
MANOVAs	multivariate analyses of variance
GLM	General Linear Model

## References

[B1-behavsci-15-00528] Amaslidou A., Ierodiakonou-Benou I., Bakirtzis C., Nikolaidis I., Grigoriadis N., Nimatoudis I. (2023). Multiple sclerosis and mental health related quality of life: The role of defense mechanisms, defense styles and family environment. AIMS Neuroscience.

[B3-behavsci-15-00528] American Psychiatric Association (2013). Diagnostic and statistical manual of mental disorders.

[B2-behavsci-15-00528] American Psychiatric Association [APA] (2000). Appendix B: Defensive functioning scale. Diagnostic and statistical manual of mental disorders.

[B4-behavsci-15-00528] Babl A., Grosse Holtforth M., Perry J. C., Schneider N., Dommann E., Heer S., Stähli A., Aeschbacher N., Eggel M., Eggenberg J., Sonntag M., Berger T., Caspar F. (2019). Comparison and change of defense mechanisms over the course of psychotherapy in patients with depression or anxiety disorder: Evidence from a randomized controlled trial. Journal of Affective Disorders.

[B5-behavsci-15-00528] Baron R. M., Kenny D. A. (1986). The moderator-mediator variable distinction in social psychological research: Conceptual, strategic, and statistical considerations. Journal of Personality and Social Psychology.

[B6-behavsci-15-00528] Békés V., Starrs C. J., Perry J. C., Prout T. A., Conversano C., Di Giuseppe M. (2024). Defense mechanisms are associated with mental health symptoms across six countries. Research in Psychotherapy: Psychopathology, Process, and Outcome.

[B7-behavsci-15-00528] Bhatt D. L., Lopes R. D., Harrington R. A. (2022). Diagnosis and treatment of acute coronary syndromes: A review. JAMA.

[B8-behavsci-15-00528] Carney R. M., Freedland K. E., Stein P. K., Watkins L. L., Catellier D., Jaffe A. S., Yeragani V. K. (2003). Effects of depression on QT interval variability after myocardial infarction. Psychosomatic Medicine.

[B9-behavsci-15-00528] Chauvet-Gelinier J.-C., Bonin B. (2017). Stress, anxiety and depression in heart disease patients: A major challenge for cardiac rehabilitation. Annals of Physical and Rehabilitation Medicine.

[B10-behavsci-15-00528] Cheng M., Zhang R., Wang M., Chang M. (2022). Relationship between cognitive emotion regulation strategies and coronary heart disease: An empirical examination of heart rate variability and coronary stenosis. Psychology & Health.

[B11-behavsci-15-00528] Chiavarino C., Rabellino D., Ardito R. B., Cavallero E., Palumbo L., Bergerone S., Gaita F., Bara B. G. (2012). Emotional coping is a better predictor of cardiac prognosis than depression and anxiety. Journal of Psychosomatic Research.

[B12-behavsci-15-00528] Collet J. P., Thiele H., Barbato E., Bauersachs J., Dendale P., Edvardsen T., Gale C. P., Jobs A., Lambrinou E., Mehilli J., Merkely B., Roffi M., Sibbing D., Kastrati A., Mamas M. A., Aboyans V., Angiolillo D. J., Bueno H., Bugiardini R., Siontis G. C. M. (2021). 2020 ESC Guidelines for the management of acute coronary syndromes in patients presenting without persistent ST-segment elevation. European Heart Journal.

[B13-behavsci-15-00528] Colovic O., Lecic Tosevski D., Mladenovic I. P., Milosavljevic M., Munjiza A. (2016). Defense mechanisms in “pure” anxiety and “pure” depressive disorders. Journal of Nervous and Mental Disease.

[B14-behavsci-15-00528] Compare A., Zarbo C., Shonin E., Van Gordon W., Marconi C. (2014). Emotional regulation and depression: A potential mediator between heart and mind. Cardiovascular Psychiatry and Neurology.

[B15-behavsci-15-00528] Cruciani G., Cavicchioli M., Tanzilli G., Tanzilli A., Lingiardi V., Galli F. (2023). Heart rate variability alterations in takotsubo syndrome and related association with psychological factors: A systematic review and meta-analysis. Scientific Reports.

[B16-behavsci-15-00528] Cruciani G., Fontana A., Benzi I. M. A., Sideli L., Parolin L. A. L., Muzi L., Carone N. (2024). Mentalized affectivity, helicopter parenting, and psychopathological risk in emerging adults: A network analysis. European Journal of Investigation in Health, Psychology and Education.

[B17-behavsci-15-00528] da Silva Machado R., Vieira I. S., Scaini C., Molina M. L., Barbosa L. P., da Silva G. D. G., Ores L., Souza L. D. d. M., Jansen K., da Silva R. A. (2023). Ego-defense mechanisms and brief psychotherapies for the management of major depressive disorder in adults: A longitudinal and quasi-experimental study. Journal of Affective Disorders.

[B18-behavsci-15-00528] DeFife J. A., Hilsenroth M. J. (2005). Clinical utility of the defensive functioning scale in the assessment of depression. The Journal of Nervous and Mental Disease.

[B19-behavsci-15-00528] Denollet J., Brutsaert D. L. (2001). Reducing emotional distress improves prognosis in coronary heart disease: 9-year mortality in a clinical trial of rehabilitation. Circulation.

[B20-behavsci-15-00528] Di Giuseppe M., Ciacchini R., Micheloni T., Bertolucci I., Marchi L., Conversano C. (2018). Defense mechanisms in cancer patients: A systematic review. Journal of Psychosomatic Research.

[B21-behavsci-15-00528] Di Giuseppe M., Conversano C. (2022). Psychological components of chronic diseases: The link between defense mechanisms and alexithymia. Mediterranean Journal of Clinical Psychology.

[B22-behavsci-15-00528] Di Giuseppe M., Lo Buglio G., Cerasti E., Boldrini T., Conversano C., Lingiardi V., Tanzilli A. (2024). Defense mechanisms in individuals with depressive and anxiety symptoms: A network analysis. Frontiers in Psychology.

[B23-behavsci-15-00528] Di Giuseppe M., Perry J. C. (2021). The hierarchy of defense mechanisms: Assessing defensive functioning with the defense mechanisms rating scales Q-sort. Frontiers in Psychology.

[B24-behavsci-15-00528] Di Giuseppe M., Perry J. C., Lucchesi M., Michelini M., Vitiello S., Piantanida A., Fabiani M., Maffei S., Conversano C. (2020). Preliminary reliability and validity of the DMRS-SR-30, a novel self-report measure based on the defense mechanisms rating scales. Frontiers in Psychiatry.

[B25-behavsci-15-00528] Di Giuseppe M., Perry J. C., Petraglia J., Janzen J., Lingiardi V. (2014). Development of a Q-sort version of the defense mechanism rating scales (DMRS-Q) for clinical use. Journal of Clinical Psychology.

[B26-behavsci-15-00528] Feng L., Li L., Liu W., Yang J., Wang Q., Shi L., Luo M. (2019). Prevalence of depression in myocardial infarction; A PRISMA-compliant meta-analysis. Medicine (United States).

[B27-behavsci-15-00528] Fiorentino F., Lo Buglio G., Morelli M., Chirumbolo A., Di Giuseppe M., Lingiardi V., Tanzilli A. (2024). Defensive functioning in individuals with depressive disorders: A systematic review and meta-analysis. Journal of Affective Disorders.

[B28-behavsci-15-00528] Fiorini Bincoletto A., Liotti M., Di Giuseppe M., Fiorentino F., Nimbi F. M., Lingiardi V., Tanzilli A. (2025). An investigation into the interplay of epistemic trust, defensive mechanisms, interpersonal problems, and sintomatology: A mediation model. Personality and Individual Differences.

[B29-behavsci-15-00528] Gallucci M. (2021). jAMM: Jamovi advanced mediation models. [jamovi module].

[B30-behavsci-15-00528] Greenberg D. M., Kolasi J., Hegsted C. P., Berkowitz Y., Jurist E. L. (2017). Mentalized affectivity: A new model and assessment of emotion regulation. PLoS ONE.

[B31-behavsci-15-00528] Greenberg D. M., Rudenstine S., Alaluf R., Jurist E. L. (2021). Development and validation of the brief-mentalized affectivity scale: Evidence from cross-sectional online data and an urban community-based mental health clinic. Journal of Clinical Psychology.

[B32-behavsci-15-00528] Hamm C. W., Bassand J.-P., Agewall S., Bax J., Boersma E., Bueno H., Caso P., Dudek D., Gielen S. (2011). ESC guidelines for the management of acute coronary syndromes in patients presenting without persistent ST-segment elevation: The task force for the management of acute coronary syndromes (ACS) in patients presenting without persistent ST-segment elevation of the european society of cardiology (ESC). European Heart Journal.

[B33-behavsci-15-00528] Høglend P., Perry J. C. (1998). Defensive functioning predicts improvement in major depressive episodes. The Journal of Nervous and Mental Disease.

[B34-behavsci-15-00528] Hyphantis T., Goulia P., Carvalho A. F. (2013). Personality traits, defense mechanisms and hostility features associated with somatic symptom severity in both health and disease. Journal of Psychosomatic Research.

[B35-behavsci-15-00528] Ibanez B., James S., Agewall S., Antunes M. J., Bucciarelli-Ducci C., Bueno H., Caforio A. L. P., Crea F., Goudevenos J. A., Halvorsen S. (2018). 2017 ESC Guidelines for the management of acute myocardial infarction in patients presenting with ST-segment elevation: The task force for the management of acute myocardial infarction in patients presenting with ST-segment elevation of the european society of cardiology (ESC). European Heart Journal.

[B36-behavsci-15-00528] Iso H., Date C., Yamamoto A., Toyoshima H., Tanabe N., Kikuchi S., Kondo T., Watanabe Y., Wada Y., Ishibashi T. (2002). Perceived mental stress and mortality from cardiovascular disease among Japanese men and women: The Japan collaborative cohort study for evaluation of cancer risk sponsored by monbusho (JACC study). Circulation.

[B37-behavsci-15-00528] Javadi B., Davazdahemamy M. H., Gheidari M. I., Noori M. (2017). The effectiveness of emotion regulation therapy (ERT) on improving depression, anxiety and stress in patients with myocardial infarction. Researcher Bulletin of Medical Sciences.

[B38-behavsci-15-00528] Jurist E. (2018). Minding emotions: Cultivating mentalization in psychotherapy.

[B39-behavsci-15-00528] Kang K., Gholizadeh L., Inglis S. C., Han H.-R. (2017). Correlates of health-related quality of life in patients with myocardial infarction: A literature review. International Journal of Nursing Studies.

[B40-behavsci-15-00528] Korkmaz H., Karasu M., Ateşçelik M., Özsoy F., Korkmaz S. (2022). The relationship between defence mechanisms and clinical variables in myocardial infarction patients. Archives of Medical Sciences. Atherosclerotic Diseases.

[B41-behavsci-15-00528] Kraiss J. T., Ten Klooster P. M., Moskowitz J. T., Bohlmeijer E. T. (2020). The relationship between emotion regulation and well-being in patients with mental disorders: A meta-analysis. Comprehensive Psychiatry.

[B42-behavsci-15-00528] Kraynak T. E., Marsland A. L., Gianaros P. J. (2018). Neural mechanisms linking emotion with cardiovascular disease. Current Cardiology Reports.

[B43-behavsci-15-00528] Krumholz H. M., Peterson E. D., Ayanian J. Z., Chin M. H., DeBusk R. F., Goldman L., Kiefe C. I., Powe N. R., Rumsfeld J. S., Spertus J. A. (2005). Report of the national heart, lung, and blood institute working group on outcomes research in cardiovascular disease. Circulation.

[B44-behavsci-15-00528] Lazzeroni D., Ciraci C., Sommaruga M., Oggioni C., Saccò M., Ziveri V., Paglialonga L., Bini M., Moderato L., Brambilla L., Coruzzi P., Cruciani G., Lingiardi V., Tanzilli A., Galli F. (2022). Perceived anxiety, coping, and autonomic function in takotsubo syndrome long after the acute event. Life.

[B45-behavsci-15-00528] Levine G. N., Cohen B. E., Commodore-Mensah Y., Fleury J., Huffman J. C., Khalid U., Labarthe D. R., Lavretsky H., Michos E. D., Spatz E. S., Kubzansky L. D., Boehm J. K., Carney R. M., Smith S. C. (2021). Psychological health, well-being, and the mind-heart-body connection a scientific statement from the american heart association. Circulation.

[B46-behavsci-15-00528] Lian Y., Xiang J., Wang X., Kaminga A. C., Chen W., Lai Z., Dai W., Yang J. (2022). Prevalence of moderate to severe anxiety symptoms among patients with myocardial infarction: A meta-analysis. Psychiatric Quarterly.

[B47-behavsci-15-00528] Lichtman J. H., Froelicher E. S., Blumenthal J. A., Carney R. M., Doering L. V., Frasure-Smith N., Freedland K. E., Jaffe A. S., Leifheit-Limson E. C., Sheps D. S. (2014). Depression as a risk factor for poor prognosis among patients with acute coronary syndrome: Systematic review and recommendations: A scientific statement from the american heart association. Circulation.

[B48-behavsci-15-00528] Liljeroos T., Humphries S., Puthoopparambil S. J., Norlund F., Olsson E. M. G. (2023). Management of emotional distress following a myocardial infarction: A qualitative content analysis. Cognitive Behaviour Therapy.

[B49-behavsci-15-00528] Liotti M., Spitoni G. F., Lingiardi V., Marchetti A., Speranza A. M., Valle A., Jurist E., Giovanardi G. (2021). Mentalized affectivity in a nutshell: Validation of the Italian version of the brief-mentalized affectivity scale (B-MAS). PLoS ONE.

[B50-behavsci-15-00528] Lo Buglio G., Cruciani G., Liotti M., Galli F., Lingiardi V., Tanzilli A. (2025). Loneliness and social isolation in individuals with acute myocardial infarction and takotsubo Syndrome: A scoping review. Healthcare.

[B51-behavsci-15-00528] Mal K., Awan I. D., Ram J., Shaukat F. (2019). Depression and anxiety as a risk factor for myocardial infarction. Cureus.

[B52-behavsci-15-00528] Malone J. C., Cohen S., Liu S. R., Vaillant G. E., Waldinger R. J. (2013). Adaptive midlife defense mechanisms and late-life health. Personality and Individual Differences.

[B53-behavsci-15-00528] Meijer A., Conradi H. J., Bos E. H., Anselmino M., Carney R. M., Denollet J., Doyle F., Freedland K. E., Grace S. L., Hosseini S. H., Lane D. A., Pilote L., Parakh K., Rafanelli C., Sato H., Steeds R. P., Welin C., De Jonge P. (2013). Adjusted prognostic association of depression following myocardial infarction with mortality and cardiovascular events: Individual patient data meta-analysis. British Journal of Psychiatry.

[B54-behavsci-15-00528] Mensour E. A., Liblik K., Mantella L. E., Johri A. M. (2022). Depressive symptoms and inflammatory markers following acute myocardial infarction: A scoping review. Health Sciences Review.

[B55-behavsci-15-00528] Moraes-Silva I. C., Rodrigues B., Coelho-Junior H. J., Feriani D. J., Irigoyen M.-C. (2017). Myocardial infarction and exercise training: Evidence from basic science. Exercise for cardiovascular disease prevention and treatment: From molecular to clinical, part 1.

[B56-behavsci-15-00528] Norekvål T. M., Fridlund B., Rokne B., Segadal L., Wentzel-Larsen T., Nordrehaug J. E. (2010). Patient-reported outcomes as predictors of 10-year survival in women after acute myocardial infarction. Health and Quality of Life Outcomes.

[B57-behavsci-15-00528] Olson T. R., Presniak M. D., MacGregor M. W. (2009). Differentiation of depression and anxiety groups using defense mechanisms. The Journal of Nervous and Mental Disease.

[B58-behavsci-15-00528] Perry J. C. (1990). The defense mechanism rating scales manual.

[B59-behavsci-15-00528] Pietrzykowski Ł., Michalski P., Kosobucka A., Kasprzak M., Fabiszak T., Stolarek W., Siller-Matula J. M., Kubica A. (2020). Medication adherence and its determinants in patients after myocardial infarction. Scientific Reports.

[B60-behavsci-15-00528] Prout T. A., Di Giuseppe M., Zilcha-Mano S., Perry J. C., Conversano C. (2022). Psychometric properties of the defense mechanisms rating scales-self-report-30 (DMRS-SR-30): Internal consistency, validity and factor structure. Journal of Personality Assessment.

[B61-behavsci-15-00528] Roest A. M., Martens E. J., Denollet J., De Jonge P. (2010). Prognostic association of anxiety post myocardial infarction with mortality and new cardiac events: A meta-analysis. Psychosomatic Medicine.

[B62-behavsci-15-00528] Rosengren A., Hawken S., Ôunpuu S., Sliwa K., Zubaid M., Almahmeed W. A., Blackett K. N., Sitthi-Amorn C., Sato H., Yusuf S. (2004). Association of psychosocial risk factors with risk of acute myocardial infarction in 11 119 cases and 13 648 controls from 52 countries (the INTERHEART study): Case-control study. The Lancet.

[B63-behavsci-15-00528] Rørholm Pedersen L., Frestad D., Mide Michelsen M., Dam Mygind N., Rasmusen H., Elena Suhrs H., Prescott E. (2016). Risk factors for myocardial infarction in women and men: A review of the current literature. Current Pharmaceutical Design.

[B64-behavsci-15-00528] Salari N., Morddarvanjoghi F., Abdolmaleki A., Rasoulpoor S., Khaleghi A. A., Hezarkhani L. A., Shohaimi S., Mohammadi M. (2023). The global prevalence of myocardial infarction: A systematic review and meta-analysis. BMC Cardiovascular Disorders.

[B65-behavsci-15-00528] Schäfer J. Ö., Naumann E., Holmes E. A., Tuschen-Caffier B., Samson A. C. (2017). Emotion regulation strategies in depressive and anxiety symptoms in youth: A meta-analytic review. Journal of Youth and Adolescence.

[B66-behavsci-15-00528] Streiner D. L. (2003). Being inconsistent about consistency: When coefficient alpha does and doesn’t matter. Journal of Personality Assessment.

[B67-behavsci-15-00528] Svedlund M., Danielson E. (2004). Myocardial infarction: Narrations by afflicted women and their partners of lived experiences in daily life following an acute myocardial infarction. Journal of Clinical Nursing.

[B68-behavsci-15-00528] Tanzilli A., Cibelli A., Liotti M., Fiorentino F., Williams R., Lingiardi V. (2022). Personality, defenses, mentalization, and epistemic trust related to pandemic containment strategies and the COVID-19 vaccine: A sequential mediation model. International Journal of Environmental Research and Public Health.

[B69-behavsci-15-00528] Tanzilli A., Di Giuseppe M., Giovanardi G., Boldrini T., Caviglia G., Conversano C., Lingiardi V. (2021). Mentalization, attachment, and defense mechanisms: A psychodynamic diagnostic manual-2-oriented empirical investigation. Research in Psychotherapy: Psychopathology, Process, and Outcome.

[B70-behavsci-15-00528] Thombs B. D., De Jonge P., Coyne J. C., Whooley M. A., Frasure-Smith N., Mitchell A. J., Zuidersma M., Eze-Nliam C., Lima B. B., Smith C. G., Soderlund K., Ziegelstein R. C. (2008). Depression screening and patient outcomes in cardiovascular care: A systematic review. JAMA.

[B71-behavsci-15-00528] Thompson D. R., Jenkinson C., Roebuck A., Lewin R. J. P., Boyle R. M., Chandola T. (2002). Development and validation of a short measure of health status for individuals with acute myocardial infarction: The myocardial infarction dimensional assessment scale (MIDAS). Quality of Life Research.

[B72-behavsci-15-00528] Thygesen K., Alpert J. S., Jaffe A. S., Chaitman B. R., Bax J. J., Morrow D. A., White H. D., Mickley H., Crea F., Van De Werf F., Bucciarelli-Ducci C., Katus H. A., Pinto F. J., Antman E. M., Hamm C. W., De Caterina R., Januzzi J. L., Apple F. S., Garcia M. A. A., Windecker S. (2019). Fourth universal definition of myocardial infarction (2018). European Heart Journal.

[B73-behavsci-15-00528] Van Beek M. H., Zuidersma M., Lappenschaar M., Pop G., Roest A. M., Van Balkom A. J., Speckens A. E. M., Voshaar R. C. O. (2016). Prognostic association of cardiac anxiety with new cardiac events and mortality following myocardial infarction. The British Journal of Psychiatry.

[B74-behavsci-15-00528] Wang L., Liu W., He X., Chen Y., Lu J., Liu K., Cao K., Yin P. (2016). Association of overweight and obesity with patient mortality after acute myocardial infarction: A meta-analysis of prospective studies. International Journal of Obesity.

[B75-behavsci-15-00528] Wen Y., Yang Y., Shen J., Luo S. (2021). Anxiety and prognosis of patients with myocardial infarction: A meta-analysis. Clinical Cardiology.

[B76-behavsci-15-00528] Wierenga K. L., Fresco D. M., Alder M., Sattar A., Moore S. M. (2022). Preliminary efficacy of an emotion regulation intervention on physical activity and depressive and anxious symptoms in individuals in cardiac rehabilitation. Journal of Cardiovascular Nursing.

[B77-behavsci-15-00528] World Health Organization (2022). World mental health report: Transforming mental health for all.

[B78-behavsci-15-00528] World Health Organization (2023). Cardiovascular diseases.

[B79-behavsci-15-00528] Yang Y., Li X., Chen S., Xiao M., Liu Z., Li J., Cheng Y. (2021). Mechanism and therapeutic strategies of depression after myocardial infarction. Psychopharmacology.

