# Supplementary material for: Depressive and Anxiety Symptoms, Defense Mechanisms, and Mentalized Affectivity in Individuals with Myocardial Infarction: An Empirical Investigation
Source: Behav Sci (Basel). 2025 Apr 14;15(4):528. doi: 10.3390/bs15040528 (PMC12024256; doi:10.3390/bs15040528)
Supplement: Supplementary file 1 [file behavsci-15-00528-s001.zip › behavsci-3493813-supplementary.pdf]

Supplementary Materials

Table S1. Defensive categories and defense levels, adapted from Perry (1990).

Defensive category	Definition
Mature defensive category (level 7)	They reflect the highest level of adaptiveness, involving defenses that integrate affects and ideas in response to conflict or stress. By allowing partial awareness of emotional and cognitive aspects, they support effective coping.
Neurotic defensive category (levels 5–6)	They reflect a middle level of adaptiveness and include defenses that manage internal or external stressors by keeping either affects or ideas out of awareness. This separation prevents the full integration of conflictual experiences, thereby avoiding overwhelming anxiety.
Immature defensive category (levels 1–4)	They reflect the lowest level of adaptiveness and inhibit awareness of unacceptable ideas or feelings, protecting the individual from perceived threats. They may operate through impulsive discharge into action, through rigid and polarized perceptions of self and others, through denial, or subtly distorting aspects of the stressors.
Defense level	Definition
Level 7: High adaptive defenses	They are effective and constructive ways to cope with stressors, involving a clear perception of such stressors and a personal commitment to adapt. Individuals aim to fulfill their motives positively while recognizing their limits and seeking support when needed.
Level 6: Obsessional defenses	They protect individuals from distressing emotions tied to certain ideas (e.g., wishes or fears) by maintaining awareness of the idea while detaching from its emotional impact. Emotions are kept out of awareness and may be expressed indirectly through generalization or contradictory statements.
Level 5: Neurotic defenses	They keep unacceptable wishes or thoughts out of awareness while allowing associated emotions to be felt. These emotions are expressed indirectly through vague or unusual clues, as the cognitive aspect of the conflict remains repressed.
Level 4: Minor image-distorting defenses	They protect the individual from experiences that threaten self-esteem (e.g., failure or criticism) by distorting certain aspects of the stressor. This temporarily supports self-image and mitigates feelings of shame or powerlessness. Unlike major image-distorting defenses, these defenses do not involve pervasive distortion.
Level 3: Disavowal defenses and autistic fantasy	They emerge when aspects of internal experience or external reality are perceived as unacceptable and thus denied. The individual avoids taking ownership of the problem, often misattributing it to external causes. This leads to a failure to recognize one's own role in the issue and limits the ability to address it.
Level 2: Major image-distorting defenses	They protect against intense anxiety triggered by conflicting representations of self or others. Positive and negative images are kept separate, leading to simplified and rigid views (e.g., all-good or all-bad). The individual relates to such distorted images accordingly, defending against fears of abandonment or annihilation.
Level 1: Action defenses	They emerge when stress or conflict is perceived as external and intolerable. Internal sources of distress—such as unacceptable wishes or fears—are overlooked, and the individual reacts by manipulating or attacking the perceived external source, leading to impulsive actions. These behaviors aim to release tension or avoid fear, without anticipating negative consequences.

Perry, J. C. (1990). The Defense Mechanism Rating Scales Manual, 5th. ed. Cambridge Hospital.

Table S2. Descriptives and differences between MI patients and healthy controls on sociodemographic and clinical information.

	MI patients (n = 67)	Healthy controls (n = 80)		
Sociodemographic information	M (SD)	M (SD)	t (df)	p
Age (years)	61.6 (9.77)	59.0 (8.46)	1.73 (145)	.086
	n (%)	n (%)	X^2(df)	p
Gender			0.99 (1)	.321
Females	16 (23.9)	25 (31.3)		
Males	51 (76.1)	55 (68.7)		
Marital status			4.85 (3)	.183
Unmarried	6 (9.0)	12 (15.0)		
Married	46 (68.7)	59 (73.8)		
Separated/divorced	7 (10.4)	6 (7.5)		
Widower	8 (11.9)	3 (3.8)		
Educational level			6.74 (4)	.150
Elementary school diploma	1 (1.5)	1 (1.3)		
Middle school diploma	17 (25.4)	8 (10.0)		
Higher school diploma	29 (43.3)	37 (46.3)		
Bachelor’s or master’s degree	17 (25.4)	29 (36.3)		

PhD or Specialization	3 (4.5)	5 (6.3)		
Clinical information	*M* (*SD*)	*M* (*SD*)	t(df)	*p*
BMI	26.6 (4.00)	24.0 (2.30)	4.92 (145)	<.001
	n (%)	*n* (%)	X^2(df)	*p*
BMI categories			26.2 (4)	<.001
Underweight	1 (1.5)	2 (2.5)		
Normal weight	21 (31.3)	52 (65.0)		
Overweight	32 (47.8)	26 (32.5)		
Obesity class I	12 (17.9)	0 (0)		
Obesity class II	1 (1.5)	0 (0)		
Smoking			63.5 (2)	<.001
No	16 (23.9)	49 (61.3)		
Yes, in the past	12 (17.9)	31 (38.8)		
Yes, currently	39 (58.2)	0 (0)		
Alcohol consumption			50.8 (2)	<0.001
No	34 (50.7)	80 (100.0)		
Yes, once or twice a week	5 (7.5)	0 (0)		
Yes, more than twice a week	28 (41.8)	0 (0)		

Dyslipidaemia			59.7 (1)	<.001
No	21 (31.3)	74 (92.5)		
Yes	46 (68.7)	6 (7.5)		
Insulinemia			9.9 (1)	.002
No	57 (85.1)	79 (98.8)		
Yes	10 (14.9)	1 (1.3)		
Pulmonary oedema			1.2 (1)	.273
No	66 (98.5)	80 (100.0)		
Yes	1 (1.5)	0 (0)		
Transient ischemic attacks			2.4 (1)	.120
No	65 (97.0)	80 (100.0)		
Yes	2 (3.0)	0 (0)		
Renal failure			2.5 (1)	.116
No	63 (94.0)	79 (98.8)		
Yes	4 (6.0)	1 (1.3)		
Chronic obstructive pulmonary disease			6.2 (1)	.013
No	62 (92.5)	80 (100.0)		
Yes	5 (7.5)	0 (0)		
Sleep apnea			2.8 (1)	.093

No	65 (97.0)	72 (90.0)		
Yes	2 (3.0)	8 (10.0)		
Neoplasms			1.1 (1)	.306
No	64 (95.5)	73 (91.3)		
Yes	3 (4.5)	7 (8.8)		
Diabetes			11.5 (2)	.003
No	57 (85.1)	79 (98.8)		
Yes, type I	1 (1.5)	1 (1.3)		
Yes, type II	9 (13.4)	0 (0)		

Note. Percentages may not equal to 100, due to rounding. *n* = number of participants, X^2 = chi-square test statistic, *t* = Student's t statistic, *df* = degrees of freedom, *p* = p value.
